# H-ferritin ferroxidase induces cytoprotective pathways and inhibits microvascular stasis in transgenic sickle mice

**DOI:** 10.3389/fphar.2014.00079

**Published:** 2014-04-17

**Authors:** Gregory M. Vercellotti, Fatima B. Khan, Julia Nguyen, Chunsheng Chen, Carol M. Bruzzone, Heather Bechtel, Graham Brown, Karl A. Nath, Clifford J. Steer, Robert P. Hebbel, John D. Belcher

**Affiliations:** ^1^Division of Hematology, Oncology and Transplantation, Department of Medicine, University of Minnesota Medical School, Minneapolis, MNUSA; ^2^Vascular Biology Center, Department of Medicine, University of Minnesota Medical SchoolMinneapolis, MN, USA; ^3^Mercy Clinic Children’s Cancer and Hematology, St. Louis, MOUSA; ^4^Division of Nephrology and Hypertension, Department of Medicine, Mayo Clinic/FoundationRochester, MN, USA; ^5^Division of Gastroenterology, Hepatology and Nutrition, Department of Medicine, University of Minnesota Medical SchoolMinneapolis, MN, USA

**Keywords:** H-ferritin, sickle cell disease, inflammation, endothelium, vaso-occlusion

## Abstract

Hemolysis, oxidative stress, inflammation, vaso-occlusion, and organ infarction are hallmarks of sickle cell disease (SCD). We have previously shown that increases in heme oxygenase-1 (HO-1) activity detoxify heme and inhibit vaso-occlusion in transgenic mouse models of SCD. HO-1 releases Fe^2+^ from heme, and the ferritin heavy chain (FHC) ferroxidase oxidizes Fe^2+^ to catalytically inactive Fe^3+^ inside ferritin. FHC overexpression has been shown to be cytoprotective. In this study, we hypothesized that overexpression of FHC and its ferroxidase activity will inhibit inflammation and microvascular stasis in transgenic SCD mice in response to plasma hemoglobin. We utilized a *Sleeping Beauty (SB)* transposase plasmid to deliver a human wild-type-ferritin heavy chain (wt-hFHC) transposable element by hydrodynamic tail vein injections into NY1DD SCD mice. Control SCD mice were infused with the same volume of lactated Ringer’s solution (LRS) or a human triple missense FHC (ms-hFHC) plasmid with no ferroxidase activity. 8 weeks later, LRS-injected mice had ~40% microvascular stasis (% non-flowing venules) 1 h after infusion of stroma-free hemoglobin, while mice overexpressing wt-hFHC had only 5% stasis (*p* < 0.05), and ms-hFHC mice had 33% stasis suggesting vascular protection by ferroxidase active wt-hFHC. The wt-hFHC SCD mice had marked increases in splenic hFHC mRNA and hepatic hFHC protein, ferritin light chain (FLC), 5-aminolevulinic acid synthase (ALAS), heme content, ferroportin, nuclear factor erythroid 2-related factor 2 (Nrf2), and HO-1 activity and protein. There was also a decrease in hepatic activated nuclear factor-kappa B (NF-κB) phospho-p65 and vascular cell adhesion molecule-1 (VCAM-1). Inhibition of HO-1 activity with tin protoporphyrin demonstrated HO-1 was not essential for the protection by wt-hFHC. We conclude that wt-hFHC ferroxidase activity enhances cytoprotective Nrf2-regulated proteins including HO-1, thereby resulting in decreased NF-κB-activation, adhesion molecules, and microvascular stasis in transgenic SCD mice.

## INTRODUCTION

Heme-driven oxidative stress and inflammation play critical roles in vaso-occlusion, endothelial cell dysfunction and chronic vasculopathy in SCD (Kato et al., 2007, 2009; Belcher et al., 2010a; Hebbel, 2011; Ghosh et al., 2013). Sickle red blood cells hemolyze, releasing hemoglobin into the vasculature, which, when oxidized to methemoglobin, can release toxic heme that promotes oxidative stress and inflammation (Jia et al., 2007; Mollan and Alayash, 2013; Schaer et al., 2013; Belcher et al., 2014). Normally, plasma hemoglobin and heme are removed safely by haptoglobin and hemopexin, but these cytoprotective scavenging proteins are depleted in the plasma of SCD patients and mice (Muller-Eberhard et al., 1968; Wochner et al., 1974; Mollan and Alayash, 2013; Vinchi et al., 2013; Belcher et al., 2014).

Transgenic sickle mice have vascular inflammation which is critical for blood cell adhesion and vaso-occlusion (Belcher et al., 2003; Kaul et al., 2004; Zhang et al., 2013). We have recently shown that free heme can activate endothelium *in vivo* and *in vitro* via the toll-like receptor 4 (TLR-4) causing Weibel-Palade body exocytosis with expression of P-selectin and von Willebrand factor on their surfaces and activation of the pro-inflammatory transcription factor NF-κB (Belcher et al., 2014). Supplemental haptoglobin or hemopexin can prevent endothelial activation and Hb/heme-induced vaso-occlusion (Schaer et al., 2013; Belcher et al., 2014). Detoxification of heme requires HO, either the inducible HO-1 or the constitutive HO-2 (Otterbein et al., 2003; Maines and Gibbs, 2005). We have shown that although HO-1 is increased in sickle patients and mice, pharmacologic or gene therapy augmentation of HO-1 activity provides protection against inflammation and vaso-occlusion in sickle mice (Nath et al., 2001; Jison et al., 2004; Belcher et al., 2006, 2010b). HO-1 degrades heme releasing Fe^2+^, carbon monoxide, and biliverdin/bilirubin. We and others have shown that CO, either inhaled or delivered by hemoglobin as MP4CO, has salutary effects in sickle mice and in human endothelial cells *in vitro* (Beutler, 1975; Belcher et al., 2006, 2013; Beckman et al., 2009). Biliverdin/bilirubin has marked antioxidant and anti-inflammatory effects, both of which are observed in SCD (Belcher et al., 2006; Kapitulnik and Maines, 2009).

Ferritin, an iron storage protein, plays an important role in iron and heme-catalyzed oxidative damage. Ferritin is composed of 24 subunits of two types: heavy (H) and light (L; Harrison and Arosio, 1996). Ferritin heavy chain (FHC) with its ferroxidase activity detoxifies heme and protects cells against heme and redox-active iron (Levi et al., 1988; Epsztejn et al., 1999; Cozzi et al., 2000; Arosio and Levi, 2002). Released Fe^2+^ from heme is oxidized via FHC ferroxidase activity, and safely stored as the catalytically inactive Fe^3+^. For several years, we have considered ferritin a cytoprotective antioxidant stratagem of the endothelium. In fact, we originally reported that FHC ferroxidase that takes up iron can protect endothelial cells against oxidative injury whereas ferroxidase-null ferritin does not (Balla et al., 1992, 2007). Multiple investigators have shown, *in vitro* and *in vivo*, that FHC can protect cells and organs (Epsztejn et al., 1999; Cozzi et al., 2000; Berberat et al., 2003; Xie et al., 2005). A recent review couples heme and iron metabolism via FHC with the pathogenesis of systemic infections and inflammation through control of labile pro-oxidant iron (Gozzelino and Soares, 2014).

The ratio of ferritin H and L subunits is tissue specific and affects iron storage and availability; this differential expression may influence heme-catalyzed oxidative damage (Harrison and Arosio, 1996). In SCD, iron overload due to enhanced iron absorption, hemolysis, and red blood cell transfusions leads to multi-organ dysfunction (Walter et al., 2009; Porter and Garbowski, 2013; Ware and Kwiatkowski, 2013). Serum ferritin, which primarily reflects apo-light chain, is increased in SCD patients and correlates with total body iron burden. Yet, for unknown reasons, levels of ferritin are insufficient to handle the catalytic heme-iron burden in SCD patients. We posited that selectively increasing FHC ferroxidase activity would provide a cytoprotective mechanism in SCD mice. However, effective means of influencing these mechanisms *in vivo* are lacking. Ferritin-inducing reagents such as heme and iron, as well as application of recombinant FHC, have been shown to protect endothelial cells from heme-peroxide challenge in cell culture (Balla et al., 1992, 2000; Lin and Girotti, 1997; Lanceta et al., 2013). Ferritin levels are controlled by cellular iron levels through a post-translational interaction with iron-response proteins 1 and 2 (IRP-1 and IRP-2), releasing these proteins from iron-binding response elements on ferritin mRNA (Rouault, 2006; Wang and Pantopoulos, 2011). Overexpression of FHC ferroxidase through transfection of a tetracycline responsive promoter or through an adenovirus had cytoprotective effects in cultured endothelial, HeLa and L929 cells; and in rat livers subjected to ischemia/reperfusion injury (Cozzi et al., 2000; Berberat et al., 2003; Xie et al., 2005). Since ischemia/reperfusion physiology underpins the pathogenesis of SCD, we hypothesized that overexpression of FHC with ferroxidase activity will attenuate hemoglobin-mediated vaso-occlusion in mouse models of SCD (Hebbel et al., 2009). We utilized a novel non-viral delivery system,* SB *transposase, for wild type (wt)-FHC and ferroxidase-null missense (ms)-FHC expression in sickle mice as previously described (Belcher et al., 2010b).

## MATERIALS AND METHODS

### CONSTRUCTION OF *SLEEPING BEAUTY *(*SB*) TRANSPOSASE/wt-hFHC AND ms-hFHC

Sticky ended directional cloning of wt human fTH-1 was completed by use of linker PCR primers. Human sequence gene specific PCR primers were designed to include unique restriction endonuclease sites also found in the pORF5 MCS and six additional nucleotides 5′ to either the start or stop codons. Total RNA was purified from a human umbilical endothelial cell preparation. Gene specific reverse transcription was followed by five cycles of touch down PCR followed by 37 cycles of PCR using the two gene specific primers (Titan one Tube, Roche). PCR product of ~584 bp was verified by agarose gel electrophoresis. Ferroxidase-null 3 ms fTH-1 (E62K, H65G, and K86Q) was created using the same primers and a PCR target template containing the 3 ms coding region (kind gift of Dr. Paolo Arosio, Università di Brescia) and a standard PCR reaction. The 3 ms fTH-1 (ms-hFHC) was used as a negative control for ferroxidase activity. pORF5-MCS (InvivoGen) and the PCR products were digested with the matching restriction endonucleases to create direction specific sticky ends. The vector and fTH-1 PCR products were cleaned to remove residual restriction endonucleases, multi cloning site fragment, and primer tail trim by size exclusion with Qiagen PCR clean up. The PCR amplified fTH-1 and pORF-MCS were ligated at 3:1 molar ends ratio overnight using T4 ligase (New England Biolabs) and then transformed into competent cells. Colonies present on ampicillin selection bacterial plates were screened for gain-of-mass and restriction mapping on agarose gel electrophoresis, ligation gap PCR reactions, and DNA was confirmed by sequencing.

pORF-MCS/human fTH-1 plasmid DNA was digested with unique restriction endonucleases AsiSi and SwaI (NEB) to release the, hEF1-eIF4g prom/human fTH-1/SV40pAn cassette from the plasmid basic elements. The hEF1-eIF4g prom/human fTH-1/SV40pAn was purified using agarose gel mass separation and PCR clean up (Qiagen), and then blunted with Klenow reaction. A blunt opening between the IR/DR sequences of the SB100X plasmid was created at the unique restriction endonuclease EcoRV site. The digested SB100X plasmid was phosphatased to reduce self religation. Blunt hEF1-eIF4g prom/human fTH-1/SV40pAn and blunt pKT2/meIF/ SB100X/pAn were ligated at 3:1 molar ends ratio overnight at 16°C and transformed into competent *E. coli*. Colonies present on kanamycin selection bacterial plates were screened for gain of mass and DNA was confirmed by Sanger sequence.

wt−hFHC…61HE**E**RE**H**AEKLMKLQNQRGGRIFLQDI**K**KPD

ms−hFHC…61HE**K**RE**G**AEKLMKLQNQRGGRIFLQDI**Q**KPD

### MICE

All animal experiments were approved by the University of Minnesota’s Institutional Animal Care and Use Committee. NY1DD mice were chosen as our sickle mouse model (Fabry et al., 1992). Equal numbers of males and females were obtained and were infused as described below at ages 8–17 weeks. The mice were housed in specific pathogen-free housing to minimize common infectious sources of inflammation and fed standard chow diet.

### GENE TRANSFER INTO MICE

DNA (25 μg) was diluted into 0.1 ml LRS/g body weight of recipient mouse, with a maximum of 2.5 ml. DNA given was either the *SB*-wt-fTH-1 or the *SB*-ms-fTH-1 DNA plasmid as described above. Vehicle control mice were injected with LRS alone. Mice were anesthetized and DNA was injected via tail veins over a course of 5–6 s. Further experiments were conducted 8 weeks after infusion to allow stable expression of resultant proteins (Belcher et al., 2010b).

### MEASUREMENT OF VASCULAR STASIS

Dorsal skin fold chambers were placed as described previously (Kalambur et al., 2004; Belcher et al., 2005). Mice recovered for three days to allow healing. At baseline, mice were anesthetized and free-flowing vessels within the visible field were identified via intravital microscopy and mapped. After baseline selection of flowing venules, mice were infused via tail vein with stroma-free hemoglobin (8 μmol/kg; a gift from Dr. Mark Young, Sangart Inc.) to simulate a hemolytic event. The same venules were re-examined 1 and 4 h after hemoglobin infusion. Venules with no observable blood flow were counted as static. The percentage of static vessels was calculated by dividing the number of static venules at 1 or 4 h by the total number of venules examined.

### HARVEST OF ANIMAL TISSUES

Blood samples and organs were then harvested 4 h after hemoglobin infusion as described previously (Belcher et al., 2005). Mice were asphyxiated in CO_2_ and blood was collected via terminal cardiac puncture. Organs were removed and processed for immunohistochemistry, RNA analysis, and homogenate preparation. Portions for immunohistochemistry were immediately dropped in phosphate-buffered formalin, while other portions were wrapped in foil, frozen in liquid nitrogen, and stored at -85^o^C.**

### mRNA ANALYSIS

Total RNA was isolated from frozen spleen sections, and human FHC and mouse HO-1 mRNAs were quantified using probe based quantitative PCR against murine GAPDH as a reference gene (Roche Applied Sciences Universal Probe Library).

### WESTERN BLOT ANALYSIS OF LIVER TISSUE FOR FHC, FLC, HO-1, Bach-1, Nrf2, NF-κB, VCAM-1, ALAS, AND FERROPORTIN

An equal amount of protein (30 μg) from liver microsomes, nuclei, or cytosol was loaded into lanes in an SDS buffer and subjected to electrophoresis on 10 or 15% polyacrylamide gels (Bio-Rad) as previously described (Belcher et al., 2005, 2013, 2014). The samples were transferred to polyvinylidene fluoride membranes (Millipore) via electrophoresis. The membranes were probed with rabbit primary antibodies against human FHC (Origene, #TA301280), mouse HO-1 (Stressgen, #OSA111), mouse NF-κB p65 (Cell Signaling #3034) mouse NF-κB phospho-p65 (Cell Signaling, #3031), mouse 5-aminolevulinic acid synthase (ALAS; GeneTex, #GTX104139), mouse ferroportin (Novus Biologicals, #NBP1-21502), mouse ferritin light chain (FLC; Origene, #TA307874), mouse VCAM-1 (Abcam, #174279), and mouse GAPDH (Sigma, #G9495). Sites of binding were visualized via the appropriate secondary IgG conjugated to horseradish peroxidase (Santa Cruz). Final detection of bands was done with ECF^ TM^ substrate (GE Healthcare) and read on a Storm^ TM^ Reader (GE Healthcare). Membranes were stripped using Restore Stripping Buffer (Thermo Scientific) and re-probed as described above.

### HO-1 ACTIVITY IN LIVER MICROSOMES

HO-1 activity was measured as previously described from fresh liver microsome preparations (Belcher et al., 2010b).

### HEME CONTENT OF LIVER MICROSOMES

Heme content was measured via the pyridine hemochromogen method (Balla et al., 1991). Briefly, liver microsomes were combined with pyridine in microcentrifuge tubes, incubated and then sodium hydroxide was added to each. Aliquots were pipetted in quadruplicate into microtiter plates. Fresh sodium hydrosulfite was added to duplicates, and potassium ferricyanide to other duplicates. The plate was read at 557 and 541 nm, the difference of the two was calculated for the oxidized and reduced samples, and the final concentration was determined by the difference between the two, expressed as μmols heme/mg protein.

### HEK-293 CELL TRANSFECTION

Human embryonic kidney (HEK-293) cells (ATCC CRL-1573) were grown in 10% FBS DMEM high glucose (Life Technologies) with 500 uM sodium pyruvate (Life Technologies), 4 mM L-Glutamine (Life Technologies), 1.5 g/L sodium bicarbonate (Sigma) and 1% antibiotic (Life Technologies). For transfection, cells were seeded overnight on glass chamber slides and transfected at 70% confluence, 2 h prior to transfection cells were washed twice with 5% FBS DMEM high glucose (Life Technologies) with 500 μM sodium pyruvate, 4 mM L-Glutamine, 1.5 g sodium bicarbonate without antibiotic. Cells were transfected with 0.1 μg of DNA/cm^2^ with human wt- and ms-fTH-1 in pORF plasmids using Lipofectamine LTX (Life Technologies) as per the instructions of the manufacturer. The growth medium was replaced 24 h post-transfection.

### IMMMUNOFLUORESCENCE

In order to investigate the cellular distribution of FHC, HEK-293 cells were fixed with 3% paraformaldehyde and permeabilized with 0.5% Triton X-100 48 h after transfection. Slides were incubated with polyclonal goat anti-FHC (Abcam, #ab80587) and then donkey cy3 (red)-conjugated anti-goat antibody (Jackson ImmunoResearch, #705–165). Slides were stained with DAPI (blue) to illuminate nuclei. Images were taken with confocal microscopy using 60× objective and merged with Adobe Photoshop^ TM^. The percentage of nuclear FHC was estimated using Photoshop by counting the number of FHC pixels co-localized with DAPI (nuclear) divided by the total number of FHC pixels in the image.

### STATISTICAL ANALYSIS

All analyses were performed using SigmaStat 2.0 (SPSS, Chicago, IL, USA). Comparison of outcomes for treatment groups was performed using one-way ANOVA.

## RESULTS

Human wt- and ferroxidase-null ms-FHC *SB* transposase DNA constructs in LRS were infused hydrodynamically into the tail veins of NY1DD sickle mice. A single death occurred due to injection-related bleeding complications. After 8 weeks, studies were performed as described below.

**FIGURE 1:**
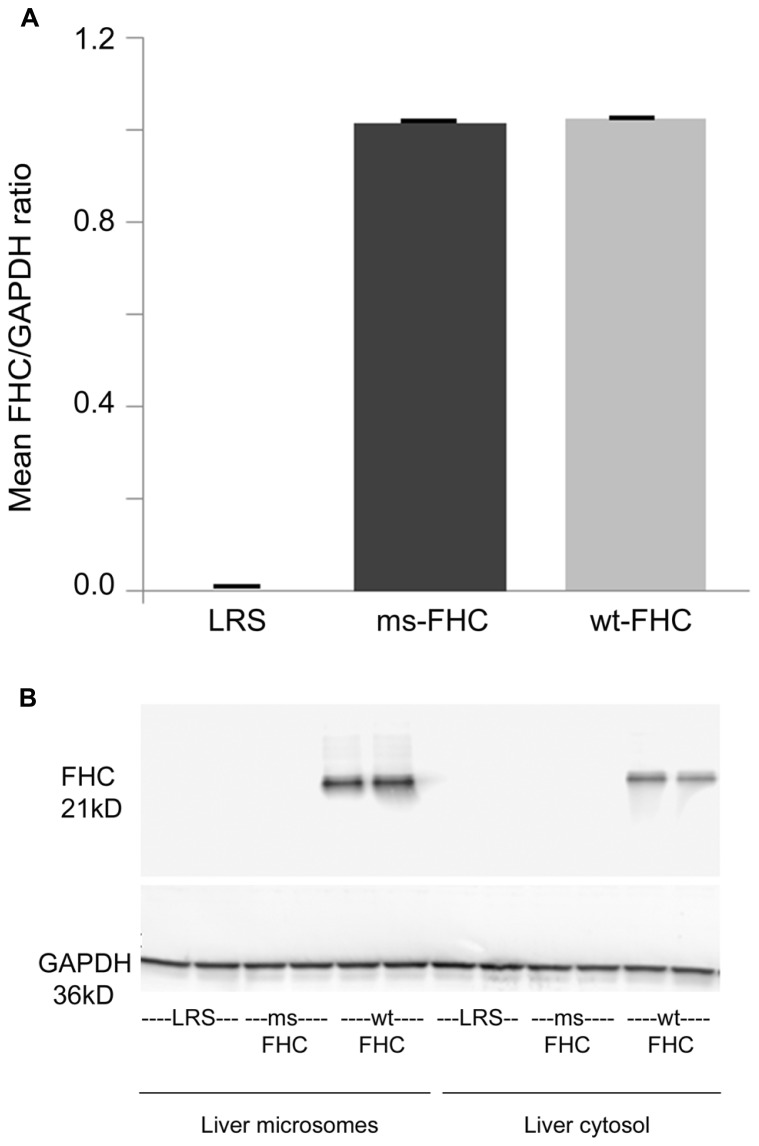
**Human ferritin heavy chain (FHC) expression is increased in transgenic sickle mice.** NYDD1 sickle mice were injected hydrodynamically with *Sleeping Beauty* (*SB)* transposase vector with either a wild-type (wt) human fTH-1 gene or a triple missense (ms) fTH*-*1 gene devoid of ferroxidase activity. Control NY1DD sickle mice were infused hydrodynamically with lactated Ringer’s solution (LRS). 8 weeks after hydrodynamic infusion, the spleens and livers were removed and flash frozen. **(A)** Total mRNA (*n *= 4 mice/treatment group) was isolated from the spleens and transcription of the human wt- and ms-fTH transgene mRNA was demonstrated by qRT-PCR, values are expressed as mean + SD of the ratio of FHC to GAPDH mRNA. **(B)** Western blots of liver microsomes and cytosol from treated sickle mice were immunostained for FHC expression. Note that because the third mutation at residue 86 is in a loop section suspected to be a conformational epitope (Addison et al., 1984), it is likely that our primary anti-FHC antibody did not bind our triple ms-hFHC protein on western blots.

Wt-hFHC and ms-hFHC mRNAs were transcribed in the spleens of the sickle mice after 8 weeks (**Figure 1A**). Wt-hFHC protein was expressed in liver microsomes and cytosol (**Figure 1B**). LRS injected animals did not demonstrate presence of human mRNA or human FHC protein (**Figures 1A,B**).

**FIGURE 2:**
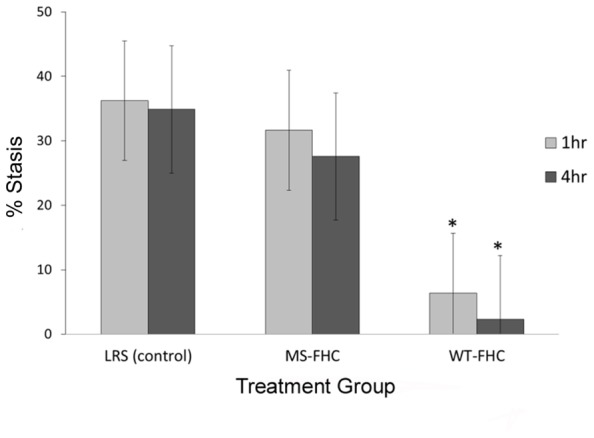
**wt-hFHC inhibits hemoglobin-induced stasis in NY1DD sickle mice.** Microvascular stasis was measured in a dorsal skin-fold chamber model 8 weeks after hydrodynamic infusion of LRS, ms-hFHC, or wt-hFHC *SB* vectors. Microvascular stasis was measured 1 and 4 h after infusion of stroma-free hemoglobin (8 μmol/kg) via the tail vein (*n* = 8 per group). Values are mean % stasis ± SD, *p  < 0.05 for wt-FHC group vs. both other groups, as calculated by one-way ANOVA.

Overexpression of FHC in NY1DD sickle mice was tested for its ability to modulate hemoglobin-induced stasis. 8 weeks after hydrodynamic infusion of LRS or *SB* FHC plasmids, dorsal skin fold chambers were placed and free-flowing venules were identified and mapped. After selection of flowing venules, stroma-free hemoglobin was infused to induce stasis. 1 and 4 h after hemoglobin infusion, the same venules were re-examined and the static venules were counted. Vascular stasis induced by hemoglobin was inhibited in the skin of sickle mice overexpressing wt-hFHC (5.2 and 2.5% at 1 and 4 h, respectively, *p * < 0.05) but not in LRS-treated sickle mice (39.8 and 45.3% at 1 and 4 h, respectively), or in sickle mice expressing ms-hFHC (33 and 27% at 1 and 4 h, respectively; **Figure 2**).

**FIGURE 3:**
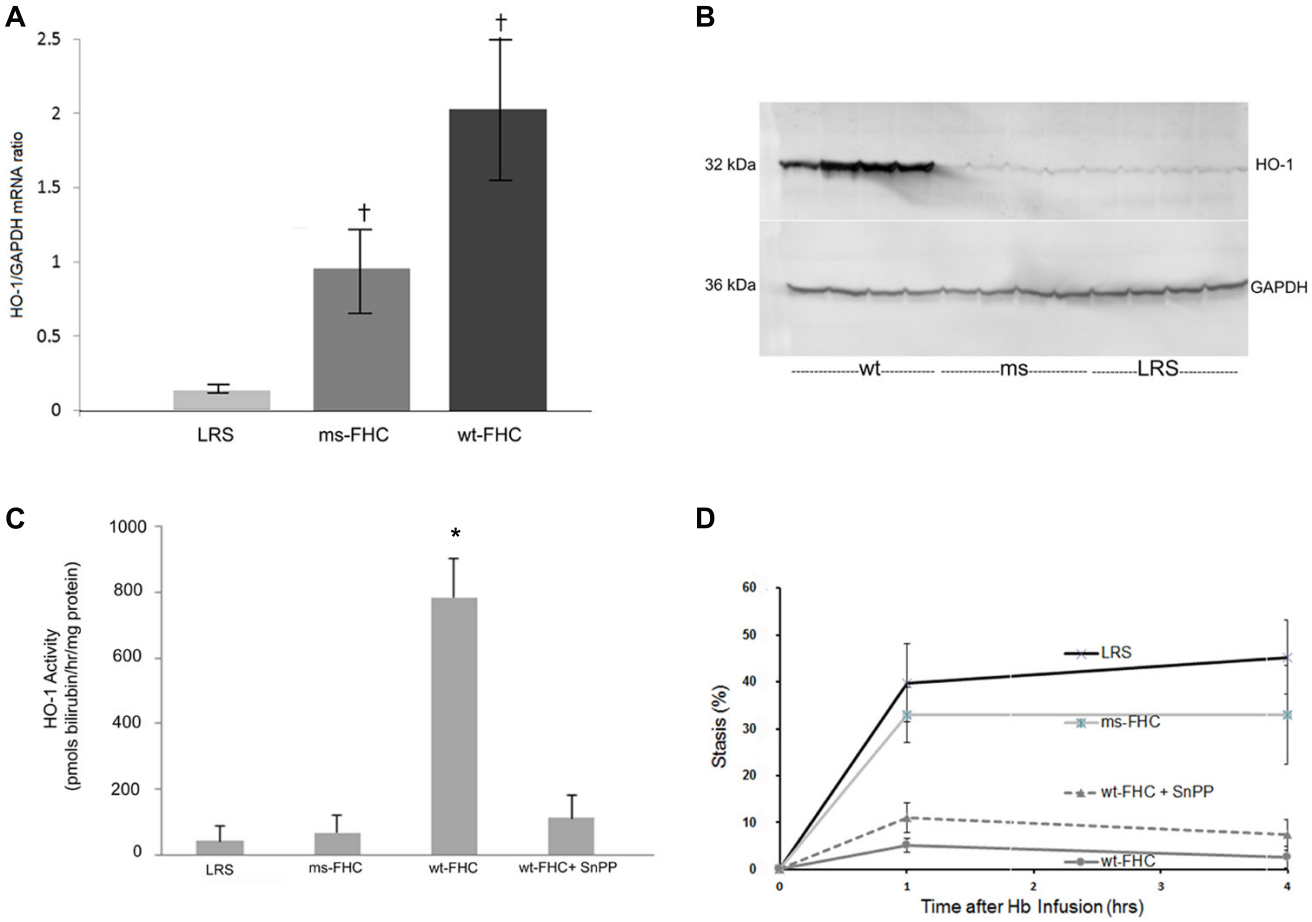
**Heme oxygenase-1 (HO-1) is up-regulated in NY1DD sickle mice expressing human wt-FHC. (A)** 8 weeks after hydrodynamic infusions, liver HO-1, and GAPDH mRNA ratios were measured by qRT-PCR (*n* = 4 mice per treatment group). Values are means ± SD, ^†^*p * < 0.05 for wt-FHC and ms-FHC vs. LRS, as calculated by one-way ANOVA. **(B)** Microsomal membranes (*n *= 4 mice per treatment group) were isolated from the livers, run on a western blot (30 μg of microsomal protein per lane), and immunostained for HO-1 and GAPDH protein expression. **(C)** Heme oxygenase (HO) enzymatic activity was measured by measuring bilirubin production using 2 mg protein of liver microsomes per reaction (*n *= 4 mice per treatment group). HO activity in mice expressing human wt-FHC treated with the HO inhibitor, tin protoporphyrin (SnPP; 40 μmol/kg/day × 3 days, intraperitoneally), was also measured. Values are means ± SD, *p  < 0.01 for wt-FHC vs. LRS, ms-FHC, and wt-FHC + SnPP, as calculated by one-way ANOVA. **(D)** Microvascular stasis was measured 1 and 4 h after infusion of stroma-free hemoglobin (8 μmol/kg) via the tail vein in mice treated with LRS, ms- and wt-FHC (*n* = 4 mice per group) as seen in Figure 2. Inhibition of HO activity with SnPP (40 μmol/kg/day × 3 days, intraperitoneally) did not block the inhibitory effect of wt-FHC on stasis. Values are mean % stasis ± SD, *p * < 0.05 for wt-FHC mice vs. LRS and ms-FHC, as calculated by one-way ANOVA.

We have previously shown that HO-1 is a modulator of inflammation and vaso-occlusion in transgenic sickle mice both by induction of HO-1 with heme as well as by HO-1 gene therapy using a *SB* transposase (Belcher et al., 2010b). In sickle mice expressing wt-hFHC or ms-FHC, there was a significant (*p * < 0.05) increase in HO-1 mRNA in the liver (**Figure 3A**). HO-1 protein was increased in liver microsomes in mice expressing wt-hFHC, but not ms-FHC or LRS injected animals (**Figure 3B**). Furthermore, there was no increase in HO-1 activity in the ms-FHC or the LRS injected animals, while wt-hFHC significantly increased bilirubin production as a measure of HO-1 activity (**Figure 3C**; *p * < 0.001); that such elevation in bilirubin arose from HO-1 was indicated by the marked reduction in bilirubin production when the competitive inhibitor of HO activity, tin protoporphyrin (SnPP), was administered intraperitoneally (40 μmols/kg) for 3 days prior to organ harvest.

Since HO-1 activity increased with wt-hFHC gene therapy, it was important to establish whether HO-1 was responsible for the inhibition of stasis as previously shown (Belcher et al., 2006, 2010b). Thus, mice injected with wt-hFHC in LRS were given SnPP intraperitoneally to block HO-1 activity. Treatment of animals overexpressing wt-hFHC with SnPP to inhibit HO-1 activity did not reverse wt-hFHC’s ability to inhibit stasis (**Figure 3D**). Therefore, wt-hFHC protects against vascular stasis in sickle mice even when HO-1 activity is blocked.

**FIGURE 4:**
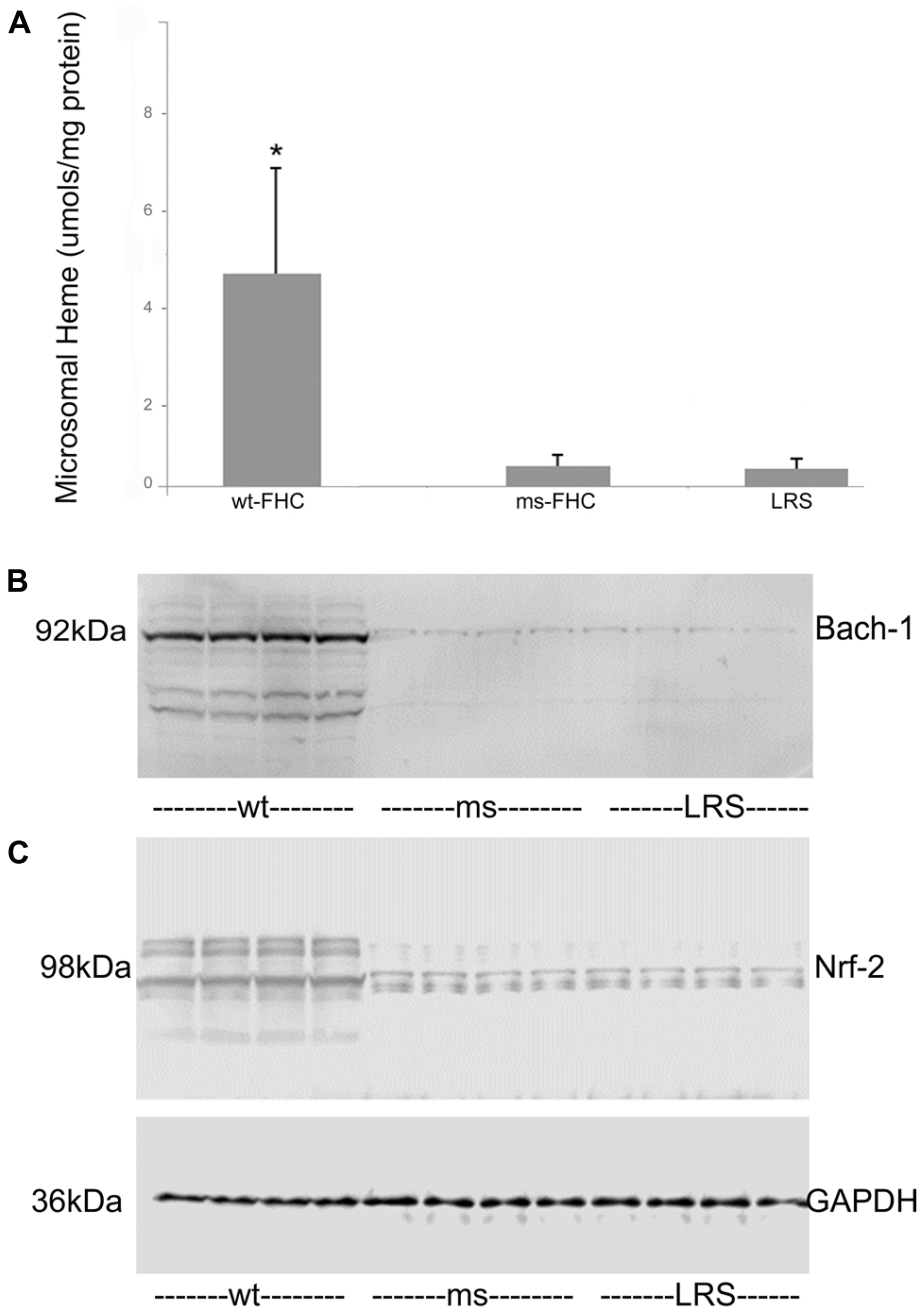
**Liver heme content and nuclear Nrf-2 and Bach-1 levels are elevated in sickle mice expressing human wt-FHC. (A)** The heme content of liver microsomes from sickle mice treated with wt-FHC, ms- or LRS was determined by the pyridine hemochromogen method (*n* = 4).Values are means ± SD, *p  < 0.05 by one-way ANOVA for wt-FHC vs. other groups. Nuclear extracts were isolated from livers, and 30 μg of nuclear extract protein from each liver was run on a western blot and immunostained for Bach-1 **(B)** and Nrf-2 **(C)**. The GAPDH loading control at the bottom applies to both **(B)** and **(C).**

To explore why HO-1 was induced by wt-hFHC, we examined whether known modulators of HO-1, such as heme, Bach-1 and Nrf-2, played a role. Heme content of liver microsomes was significantly increased in wt-hFHC mice compared to ms-FHC or LRS mice (*p* < 0.05; **Figure 4A**). Surprisingly, nuclear Bach-1 and Nrf-2 that the regulate HO-1 expression negatively and positively, respectively (Sun et al., 2004; Liu et al., 2013), were both increased in the nuclei of wt-hFHC expressing mice compared to LRS- or ms-hFHC-treated mice (**Figures 4B,C**).

**FIGURE 5:**
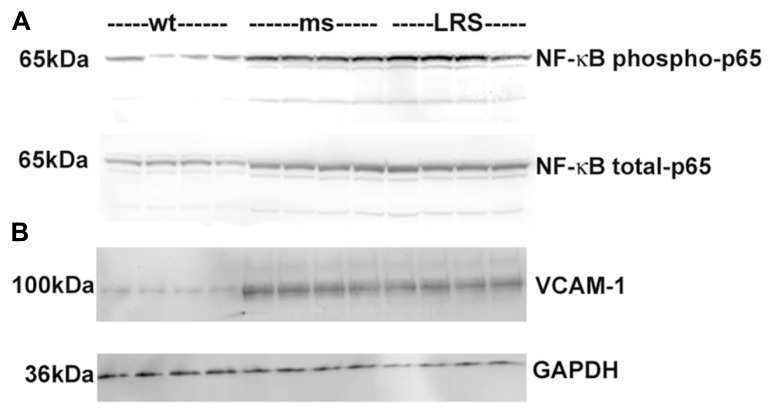
**Decrease in nuclear phospho- and total NF-κB p65 and VCAM-1 in sickle mice overexpressing wt-FHC. (A) **Nuclear extracts were isolated from livers, and 30 μg of nuclear extract protein from each liver was run on a western blot and immunostained for phospho- and total NF-κB p65 in wt-, ms-, and LRS-treated mice (*n* = 4). **(B)** Microsomal membranes were isolated from livers, and 30 μg of microsomal protein from each liver was run on a western blot and immunostained for VCAM-1.

To determine the basis for the protective effects of wt-hFHC on hemoglobin-induced stasis in SCD, we considered the involvement of NF-κB-mediated adhesion molecule expression. We have previously shown that NF-κB activation plays a critical role in vascular inflammation, endothelial cell adhesion molecule expression and blood cell adhesion in sickle mice. In an NF-κB p50 knockout bred into sickle NY1DD mice, hemin did not induce stasis, compared to wt sickle mice (Kollander et al., 2010). Animals that received gene therapy with wt-hFHC had diminished hepatic NF-κB activation as evidenced by significantly (confirmed by densitometry, data not shown) decreased phosphorylation of NF-κB p65 in the nucleus compared to ms-FHC or LRS injected animals (**Figure 5**). Similarly, VCAM-1 is an NF-κB-driven adhesion molecule that is required for vaso-occlusion in SCD mice and was decreased in liver microsomes of animals overexpressing wt-hFHC compared to animals treated with ms-hFHC or LRS (**Figure 5**).

**FIGURE 6:**
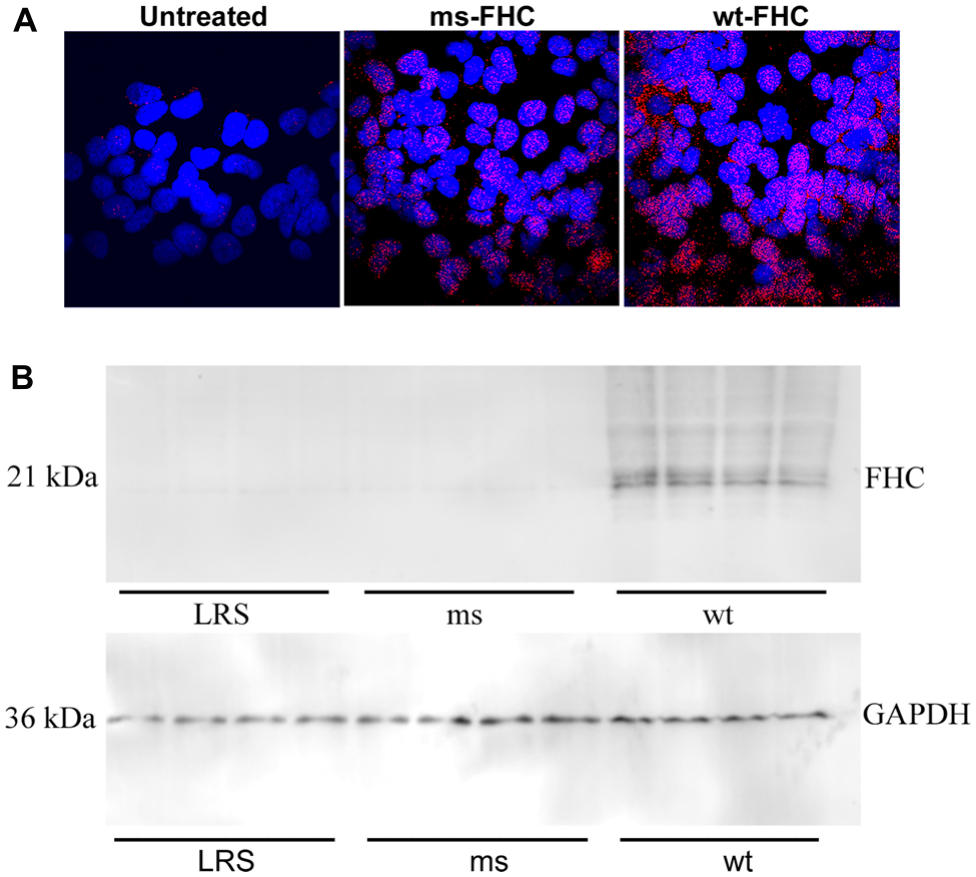
**Human FHC is expressed in the nuclei of mice expressing wt- and ms-FHC. (A)** Immunofluorescence for human FHC (red) in HEK-293 cells untreated or transfected with wt-FHC or ms-FHC constructs. Nuclei were stained with DAPI (blue). **(B)** Nuclear extracts from the livers of LRS-, ms-, and wt-FHC treated mice (*n* = 4) were isolated; 30 μg of nuclear extract protein from each liver was run on western blot and immunostained for human FHC. Note: different primary antibodies to FHC were used for **(A)** and **(B)**.

Ferritin has been implicated as a nuclear transcription factor for a variety of genes (Cai et al., 1998; Pountney et al., 1999; Alkhateeb and Connor, 2010) and could potentially affect NF-κB activation. Thus, we examined nuclear FHC localization by immunofluorescence in HEK-293 cells transfected with wt- or ms-hFHC. There were marked increases in hFHC expression in cells transfected with wt- and ms-hFHC compared to untreated cells (**Figure 6A**). In untreated cells ~68% of the FHC was co-localized with the nuclear DAPI stain. Nuclear hFHC expression was 90 and 96% in cells transfected with wt-hFHC and ms-hFHC, respectively. In SCD mice, liver nuclear extracts demonstrated significantly increased nuclear hFHC protein on western blot in mice overexpressing wt-hFHC relative to mice treated with ms-hFHC or LRS (**Figure 6B**). The difference in ms-hFHC expression seen by immunofluorescence (**Figure 6A**, non-denatured protein) and western blot (**Figure 6B**, denatured protein) may be due to differences in antibody preparations used and antibody recognition of triple ms-hFHC and wt-hFHC under denaturing and non-denaturing conditions.

**FIGURE 7:**
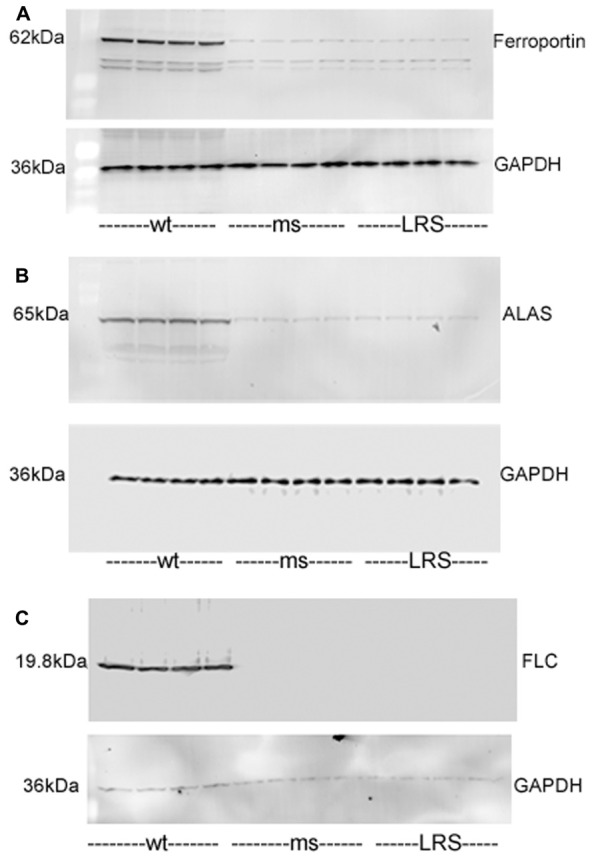
**Mouse ferroportin, 5-aminolevulinic acid synthase (ALAS), and ferritin light chain (FLC) proteins are increased in sickle mice overexpressing human wt-FHC.** Proteins of subcellular fractions isolated from livers of wt-, ms-, and LRS-treated mice (*n* = 4) were run on a western blot (30 ug protein/lane) and immunostained for **(A)** microsomal ferroportin, **(B)** mitochondrial ALAS, and **(C)** cytosolic FLC.

We examined whether other proteins involved in iron or heme metabolism were affected by increases in wt-hFHC. Ferroportin, ALAS and FLC were markedly increased in the livers of SCD mice overexpressing wt-hFHC compared to mice treated with ms-hFHC or LRS (**Figures 7A–C**). All three iron-related proteins were markedly increased in mice treated with wt-hFHC compared to mice treated with ms-hFHC or LRS.

## DISCUSSION

Our results indicate that overexpression of wt-hFHC decreases NF-κB activation and VCAM-1 expression, increases HO-1 expression, and attenuates hemoglobin-mediated vaso-occlusion in SCD mice. Furthermore, the ferroxidase activity of FHC was essential to this protection, as ms-hFHC did not protect.

We and others have previously shown that anti-inflammatory therapies or treatment with antibodies targeting adhesion molecules, including VCAM-1, E-selectin, ICAM-1, P-selectin, α4β1, αVβ3, vWF, or PECAM-1, inhibit vaso-occlusion in SCD mice (Kaul et al., 2004; Belcher et al., 2005, 2006, 2010b, 2013, 2014). Therefore, it is not surprising that decreases in inflammatory tone as reflected by lower NF-κB activation and VCAM-1 expression in SCD mice overexpressing wt-hFHC were accompanied by declines in vascular stasis. However, the mechanism of FHC anti-inflammatory action remains speculative.

Hemoglobin S is known to auto-oxidize 1.7-fold faster than hemoglobin A resulting in a higher rate of conversion to methemoglobin and generation of superoxide (·O_2_^-^) radicals and subsequent amplification of oxidative stress (Hebbel et al., 1988). To that end, scavenging free hemoglobin and heme with haptoglobin and hemopexin, two plasma proteins depleted in hemolyzing sickle cell patients, is a critical defense for the vasculature in SCD. We and others have shown the beneficial effects of supplemental haptoglobin or hemopexin in states of free hemoglobin or free heme excess (Schaer et al., 2013). Detoxifying heme through the induction of HO-1 is another stratagem that can provide vasculoprotection in SCD (Belcher et al., 2006, 2010b).

A clear protective benefit has been shown with upregulation, either pharmacologically or by gene therapy, of the HO-1 system in murine models of SCD. In SCD patients, individuals with a short GT nucleotide repeat in the *hmox-1* gene promoter, hence greater levels of inducible HO-1 protein, had fewer hospitalizations for acute chest syndrome (Bean et al., 2013). Furthermore, the products of HO-1 including carbon monoxide and biliverdin decrease vaso-occlusion in sickle mice (Belcher et al., 2006). Iron is released from heme by HO-1 and ultimately placed as Fe^3+^ in the core of ferritin which can safely store ~4500 atoms of iron. Ferritin’s role in SCD is usually considered malevolent as elevated serum ferritin reflects iron overload. Ferritin’s potential cytoprotective role in SCD has not been explored.

The basis for the present study on ferritin in SCD was initially published in 1992 (Balla et al., 1992). We had shown that heme, markedly aggravated endothelial cytotoxicity engendered by oxidants. In contrast, however, if cultured endothelial cells were briefly pulsed with heme and then allowed to incubate for a prolonged period (16 h), the cells became highly resistant to oxidant-mediated injury and to the accumulation of endothelial lipid peroxidation products. This protection was associated with the induction for both HO-1 and ferritin. Differential induction of these proteins suggested that ferritin was the primary cytoprotectant. A site-directed mutant of ferritin (heavy chain Glu62---Lys; His65----Gly), which lacks ferroxidase activity and is deficient in iron sequestering capacity, was completely ineffective in protecting cells from injury.

In these studies, FHC was designed to integrate ubiquitously using an *SB100X* transposase driven by a constitutive hEF1-eIF4g promoter. The transposase, delivered in *cis* with the wt- and ms-fTH-1 transgene, binds to IR/DR sites flanking the transgene, and catalyzes the excision of the flanked transgene, mediating its insertion into the target host genome with an apparently equal preference for AT-rich dinucleotide insertion sites in introns, exons, and intergenic sequences (Vigdal et al., 2002). We looked primarily at the liver and spleen in these animals but did not attempt to identify and characterize specific integration sites. Transient (~4–6 weeks) episomal expression of the transgene can also occur in organs.

As can be seen in **Figure 1**, the spleen and liver showed evidence for integration of the hFHC genes eight weeks after infusion, with increased levels of mRNA in the spleen and expression of wt-hFHC protein observed in the liver. Most notably, sickle animals expressing wt-hFHC were resistant to hemoglobin-induced stasis (**Figure 2**), while ms-hFHC- or LRS-treated animals had significant vaso-occlusion.

In this study, HO-1 was up-regulated at both the transcriptional and translational levels in mice treated with wt-hFHC. This co-expression of FHC and HO-1 suggests intertwined physiology in cytoprotection rather than independent pathways. However, in the study published by Balla et al. (1992), inhibition of HO-1 with SnPP in endothelial cells overexpressing wt-FHC did not reverse the protection afforded by ferritin. A residual effect of endogenous CO produced by HO-1 has not been ruled out. CO has been shown to modulate TLR4 responses in acute pancreatitis (Xue and Habtezion, 2014) and possibly responses to heme in sickle mice. Overexpression of HO-1 or administration of exogenous CO decreases inflammatory tone assessed by NF-κB activation. The decrease in NF-κB activation in wt-hFHC mice (**Figure 5**) could in part be related to increased HO-1 or through anti-oxidative effects of the ferroxidase activity of wt-hFHC.

Our initial studies demonstrating the cytoprotective properties of HO-1 emphasized the attendant induction of ferritin as a necessary accompaniment for HO-1 to confer its protection (Nath et al., 1992). Our prior studies demonstrating the protective effects of ferritin in the heme-exposed endothelium revealed that ferritin can elicit cytoprotection without the concomitant need for intact HO activity (Balla et al., 1992). Our present studies thus complete the loop by demonstrating that ferritin itself feeds back to induce HO-1, but such induction of HO-1 is not essential for ferritin to evince its protective effects. This latter finding is germane to recent observations in a mutant murine model in which H-ferritin is conditionally deleted in the renal proximal tubule; such mice are markedly sensitive to acute kidney injury, despite exhibiting higher expression of HO-1 in the kidney (Zarjou et al., 2013). Thus induced HO-1 may not prove protective if ferritin is absent, and ferritin can exert protection even in the absence of functional HO activity.

Bach-1 was increased in liver nuclear extracts from mice overexpressing wt-hFCH (**Figure 4B**) despite the marked increases in HO-1 (**Figures 3A–C**). Bach-1 and small Maf heterodimers that bind to Maf recognition elements (MARE) repress HO-1 transcription. Heme binds to Bach-1 and displaces Bach-1/Maf heterodimers from MARE allowing Nrf2 and other transcription factors to bind and induce HO-1 transcription. The displaced Bach-1 is normally exported from the nucleus and degraded. The elevated nuclear Bach-1 levels, in the face of marked increases in Nrf2 and HO-1, could possibly be related to intracellular transport or new production of heme. Along with heme (**Figure 4A**), ALAS, the rate-limiting enzyme in heme biosynthesis was increased in mice overexpressing wt-hFHC (**Figure 7B**). It is possible the wt-hFHC mice increased heme biosynthesis with altered trafficking of heme/iron in the cell. In animals treated with wt-FHC, significantly higher heme loads and Nrf2 could potentially overwhelm Bach-1 leading to induction of other cytoprotective molecules. Alternatively, nuclear FHC could prevent the phosphorylation of Bach-1. Antioxidant-induced phosphorylation of tyrosine 486 on Bach-1 is essential for the nuclear export of Bach-1. Perhaps formation of heme/Bach-1 complexes, even without nuclear export and degradation, may be sufficient to inhibit the formation of Bach-1/small Maf heterodimers and binding to MARE.

Direct nuclear effects of FHC have been described (Pountney et al., 1999; Surguladze et al., 2004; Alkhateeb and Connor, 2010). FHC was protective of DNA from UV damage in the corneal lens cells (Cai et al., 1998). A DNA-binding motif in H-ferritin raised the novel possibility of a role for ferritin as a conventional transcription factor associated with the beta-globin locus promoter (Broyles et al., 2001; Alkhateeb and Connor, 2010). We found wt-hFHC in the nuclei of HEK-293 transfected cells and in nuclear extracts of liver cells *in vivo* (**Figures 6A,B**). The effect of FHC in the nucleus appears to be specific to the heavy chain, in part because the light chain is undetectable (Alkhateeb and Connor, 2010). We found increased expression of stress-responsive proteins Nrf-2 and HO-1 in mice overexpressing wt-hFHC raising the question whether nuclear ferritin may play a role in driving an anti-inflammatory cassette of genes. We found increased ferroportin which could drive iron out of cells, light chain ferritin to store iron and ALAS to utilize iron in the mitochondria, findings that are all in support of this possibility (**Figure 7**). As elegantly recently proposed, FHC can have remarkable effects in a variety of inflammatory states due to its ability to metabolically adapt tissues to iron overload (Gozzelino and Soares, 2014).

In conclusion, our results support the notion that overexpression of FHC ferroxidase protects sickle mice from injury associated with heme-mediated vaso-occlusive disease. Although ferritin has been posited as cytoprotective for decades, upregulation of FHC has proven a challenge especially in sickle patients. As with HO-1, clearly ferritin is more friend than foe in hemolytic disease. Newer therapeutics, including gene therapy approaches may prove to be a valuable asset to the management of SCD.

## AUTHOR CONTRIBUTIONS

Gregory M. Vercellotti designed the research, analyzed data, and wrote the paper. Fatima Khan performed research and wrote the paper. Heather Bechtel performed research and analyzed data. Karl A. Nath designed experiments and wrote the paper. Julia Nguyen performed the research and analyzed the data. Chunsheng Chen performed the research and analyzed the data. Carol Bruzzone performed the research, analyzed the data, and wrote the paper. Graham Brown performed and designed experiments and analyzed data. Clifford J. Steer designed the research, analyzed the data, and wrote the paper. Robert P. Hebbel designed experiments and wrote the paper. John D. Belcher designed the research, analyzed data, and wrote the paper.

## Conflict of Interest Statement

The authors declare that the research was conducted in the absence of any commercial or financial relationships that could be construed as a potential conflict of interest.
